# Product News

**DOI:** 10.1155/S1463924682000248

**Published:** 1982


### Product News

### New ion-selective meter, UV/VIS spectrophotometer, and atomic absorption spectrophotometer

Pye Unicam, which is a part of Philips' Science and Industry Product Division, announced three new instruments to the technical press in February.

### *PW 9415 ion-selective meter*

The first to be discussed was a microprocessor-controlled ion-selective meter which is priced to be within reach of most laboratories. The meter allows direct measurement of mV, pH, pX, °C and concentration; it is also possible to use the incremental methods—standard addition and subtraction, sample addition and subtraction. A special low-level concentration mode means that results can be obtained even when the electrode has a non-linear response. The PW 9415 has a seven-segment, six-digit LED display which shows results clearly and takes the operator through the measurement sequence with a series of easily recognized prompts. Results can be reprocessed on a printer or computer if required. Other standard features include an anti-spill membrane keyboard, self-check and diagnostic capability, and analogue output for a recorder.

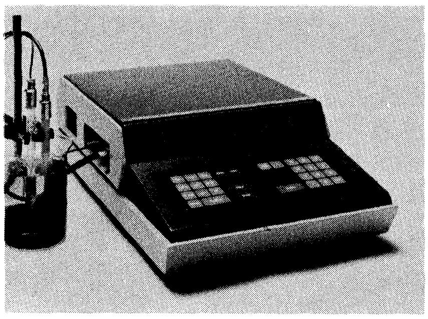

*PW 9415 ion-selective meter (Pye Unicam Ltd, Cambridge).*

**Circle No. 4 on Reader Enquiry Card**

### *PU 8800 UV visible spectrophotometer*

The PU 8800, available in single or double monochromator form, features video display of instrument status, data, graphics and operator prompts. The spectrophotometer uses the company's master holographic grating rather than replicas and replaces the SP8-200 and SP8-250 instruments in Pye Unicam's range. A self-test program calibrates the instrument to deuterium-emission lines, checks absorbance read-out against three filters, display lamp age/energy and indicates the nature and location of electronic, optical and mechanical faults. A laboratory's 10 most commonly used programs can be memorized with their titles for ease of identification and recall. The wavelength scanning facilities include a unique mode, called Synchroscan +, which, after an 'Auto' scan speed is selected, speeds through flat areas of the spectrum and slows down for peaks, ensuring that they are neither shifted nor cropped. The wavelength and value of each peak or trough detected can be displayed and/or printed. The PU 8800 has an optional printer which can be used to obtain hard copy of any video-screen display. A single print-out can list instrument settings, date, operator code, assay title, each data point and a plot of the data. A bi-directional RS 232C is standard and various software packages for programmable calculators will permit, for example, colour scanning. Other standard features include 1st to 4th derivative and log A scanning, digital smoothing, wavelength selection and reaction kinetics.

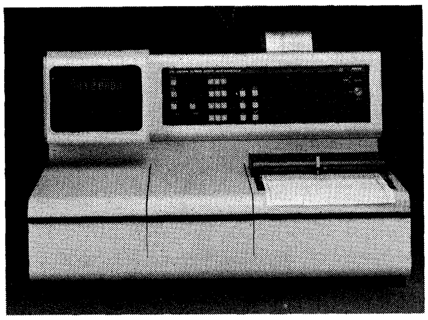

*U/V spectrophotometer with status display and graphic print-out (Pye Unicam, Cambridge).*

**Circle No. 5 on Reader Enquiry Card**

### *PU 9000—advanced multi-element AA system*

Possibly the most exciting instrument announced at Pye Unicam's press conference was the PU 9000—a fully automatic sequential atomic absorption system. This is the first intelligent AA system on the market and is capable of selecting and optimizing the conditions for each element during an automatic multi-element run. Thus it is not necessary for the operator to have any prior knowledge of conditions for any elements in a run. The instrument can, if necessary, be manually overriden. Two new developments, which help to make full use of the machine's intelligence, are a data coding system on the hollow cathode lamps (the element and maximum current for each lamp can be detected automatically); and newly designed control systems (conventional rotary gas-control valves, for example, have been replaced by a binary gas-flow switching system).

The PU 9000 has a new optical system which overcomes the limitations of single- and double-beam designs. The system has

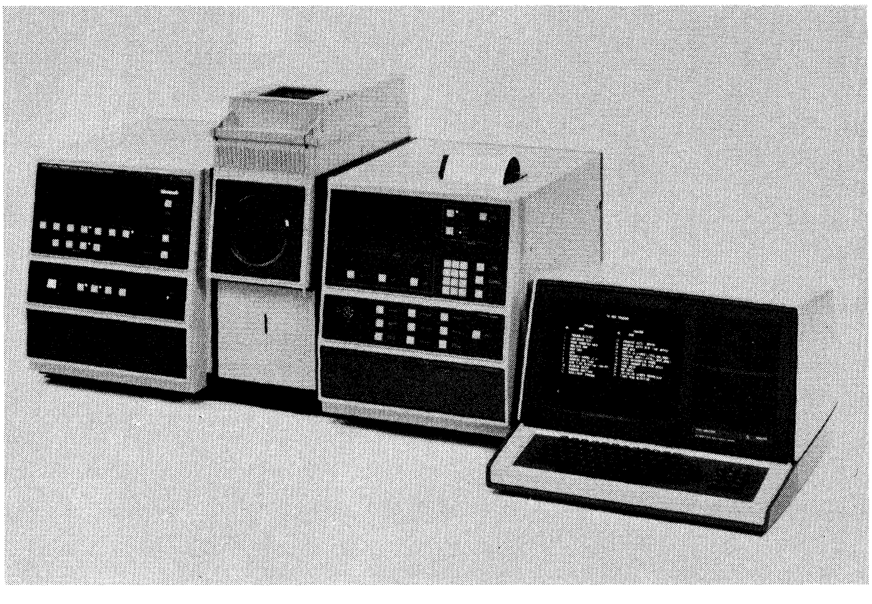

*PU 9000 atomic absorption spectrophotometer (Pye Unicam Ltd, Cambridge).*

double-beam stability without the degradation of signal-to-noise ratio found in conventional double-beam systems. Full energy is transmitted during measurement and this, combined with a master (not a replica) holographic grating, gives unprecedented signal-to-noise ratios. As a result of the optical system, and advanced signal-handling electronics, the instrument has a background correction capability of over 2A even with fast transient signals.

The PU 9007 data/control station has been developed for this AA system and it allows full external computer control for up to 16 elements and offers a number of built-in facilities. These include, for example, video display of programs, task sequences, transients, calibration, ash/atomize curves and autosampler loading guide. Twin floppy discs give fast operation and storage of displays, programs and results. Hard copy of any video display can be obtained from a built-in printer/plotter. The data/control station can be programmed in BASIC or any other common high-level language so the system can be easily modified to meet special requirements.

The PU 9000 series is available in a variety of configurations, for example without operator over-ride. A built-in autosampler is another option as is the PU 9095 video graphite furnace system and furnace autosampler.

*Further information from Pye Unicam Ltd, York Street, Cambridge CB1 2PX, UK. Tel.: 0223 358866.*

**Circle No. 6 on Reader Enquiry Card**

### Mass spectrometer—Micromass 707E

The Micromass 7070E, a mass spectrometer made by VG Analytical Ltd, was demonstrated at the Pittsburgh Conference. The new machine is intended to set new standards for magnetic sector instruments in terms of scan rates and minimum cycle times, whilst having the mass range and sensitivity required for successful FAB (fast atom bombardment) analysis.

VG Analytical have also just published a booklet called *A Review of Recent Applications of the Fast Atom Bombardment Source* which contains 30 examples of FAB spectra obtained with Micromass instruments. The pamphlet is intended for mass spectroscopists who are still evaluating the technique.

*Further information from David Parsons, VG Analytical Ltd, Tudor Road, Altrincham, Cheshire WA14 5RZ, UK. Tel.: 061 928 6300.*

**Circle No. 7 on Reader Enquiry Card**

### Water analyser

The PTI Digital Water Analyser measures pH, conductivity, mV and temperature, thus combining three instruments into one. The instrument's pH section provides 0·01 pH precision over the full range from 0 to 14 pH, it also provides 1 mV resolution for redox or ion-selective electrode measurements. The analyser has an isopotential control,

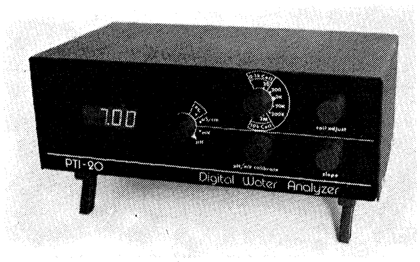

*The PTI Digital Water Analyser, which measures pH, conductivity, temperature and mV. (Aqua Chemical Company, Dover, UK.)*

which allows initial buffer calibration of the instrument and electrode system over the range of pH 6 to pH 8—most pH meters use a conventional pH 7 zero point. Slope control allows corrections to be made for electrode output differences to ensure accuracy. Temperature compensation can be automatic with an optional temperature probe which measures to ±0·1°C from −30°C to 105°C; glass and stainless-steel temperature probes are available.

The analyser's conductivity section gives direct readings of solution conductance and, if used with a glass/platinum conductivity cell, will cover the range from 0·1 to 2000 $\mu$S/cm. The range can be extended with different cells to 0·01 $\mu$S/cm and 2 S/cm.

The analyser can simultaneously measure pH, conductivity and temperature; each parameter can be displayed and, if necessary, recorded.

*Further information from Aqua Chemical Company, 166 Snargate Street, Dover, Kent CT17 9BZ, UK. Tel.: 0304 212269.*

**Circle No. 8 on Reader Enquiry Card**

### Micro-controlled amino-acid analysis

A micro-controlled amino-acid analyser, called the Alpha, has been developed by LKB Biochrom Ltd. The Alpha's micro will store up to 20 different running programs, each of which can include up to 20 individual steps—the programs can be listed so that samples may be run in sequence under varying operating conditions. Samples are loaded in capsules in an integral refrigerated loader and between 5 and 160 $\mu$l can be used—this means that the optimal sample volume can be loaded and analysed. A unique feature of the Alpha is a heated glass column, which gives the instrument the advantages of clear visibility of the resin bed and solid-state heating: there is no need for circulating water-baths.

The Alpha has excellent resolving power and a single, low-volume, long-pathlength flow-cell ensures minimal peak diffusion and extra sensitivity. A fluorescence detector is available which can enhance the sensitivity of the Alpha by a factor of 10 compared to ninhydrin detection.

*Information on the amino-acid analyser can be obtained from Mr Phil Morris, LKB Instruments Ltd, 232 Addington Road, Selsdon, South Croydon CR2 8YD, UK. Tel.: 01 657 8822.*

**Circle No. 9 on Reader Enquiry Card**

### Sour gas regulators

Stainless-steel fluid control components have been demonstrated to stress-crack in the presence of hydrogen sulphide. As a result, the US National Association of Corrosive Engineers (N.A.C.E.) have published stringent specifications for materials which should be used to contain and control sour gas under pressure—the specifications pay attention to a choice of compatible materials, to grades of steel and to the hardness of the steel used. A range of pressure regulators, including both the diaphragm and piston type of hand-loaded pressure reducers and back-pressure regulators, which meet or exceed the N.A.C.E. specifications are made by Tescom. Tescom's 44-1100 series is designed for a reduction of primary pressure up to 10 000 psi. A choice of six outlet pressure ranges is available in this series. Lower inlet pressures can be controlled with the 26-1500 series, these are diaphragm-type regulators and there is a choice of three outlet pressure ranges. Tescom's 26-1700 series is intended for controlling back pressures up to 10 000 psi, there is a choice of seven controlled pressure ranges in this set.

Tescom regulators are already used in a number of gas-control systems, both on- and offshore. Typical applications include the supply of gas to analytical and monitoring equipment.

*The regulators are being distributed in the UK by Techmation Ltd, 58 Edgware Way, Edgware, Middlesex HA8 8JP, UK. Tel.: 01 958 3111.*

**Circle No. 10 on Reader Enquiry Card**

### 'Industrial Robots Industry in Japan'

A report compiled by the Yano Economic Research Institute on the Japanese industrial robot industry will be available later this year. The report is divided into four parts: 'the industrial circle and its present situation'; 'medium-term prospects for the industrial robot market'; 'actual conditions of industrial robots by functions'; and a 'profile of 50 major robots manufacturers'. The report projects that the value of robots made in Japan will reach 255 billion yen in 1984; 20 billion yen's worth of these will be exported.

*The report costs $426, plus $20 air-mail postage, and is available from Information Researchers Inc., No. 59–3, Yoyoyi 4-chome, Shibuya-ku, Tokyo 151, Japan.*

**Circle No. 11 on Reader Enquiry Card**

### Liquid handling

Dilutrend is a new computer diluter from the Boehringer Corporation. The instrument can handle volumes between 1 μl and 10 ml at a selectable speed of operation between 2 s and 15 s. The number of consecutive operations can be preselected. All functions are controlled by the microprocessor and the user is 'piloted' by optical and acoustic signals. Frequently used programs can be stored in the memory and can be recalled with a minimum of key operations. Applications include dispensing (all liquids except hydrogen fluoride and its compounds); double dispensing; diluting (volumes for a given ratio or mixture are calculated automatically); pipetting; sample distribution (Dilutrend can aspirate 1 ml and then dispense 10 μl, 20 μl, 30 μl and 40 μl in sequence); and titration (reagents can be added continuously or in steps).

*Further information from the Customer Service Department, The Boehringer Corporation (London) Ltd, Bell Lane, Lewes, East Sussex BN7 1LG. Tel: 07916 71611.*

**Circle No. 12 on Reader Enquiry Card**

### Spectrophotometer for research

Varian have announced a new spectrophotometer which will speed and simplify analysis. The UV-VIS-NIR (near infrared) spectrophotometer, part of their 2300 series, has exceptional photometric performance in the range 3152 to 185 nm. A revolutionary design using double-pass, double-sided diffraction grating provides analytical power from the ultra-violet to infra-red range. The machine is dual-microprocessor controlled and includes a CRT display, which guides the operator through set-up; analytical methods can be stored on cassette. The spectrophotometer is suitable for a wide range of applications—quality control to drug analysis, biochemical and industrial research.

*Further information from Varian Associates Ltd, 24–28 Manor Road, Walton-on-Thames, Surrey, UK, or Varian AG, Steinhauserstrasse, CH-6300 Zug, Switzerland.*

**Circle No. 13 on Reader Enquiry Card**

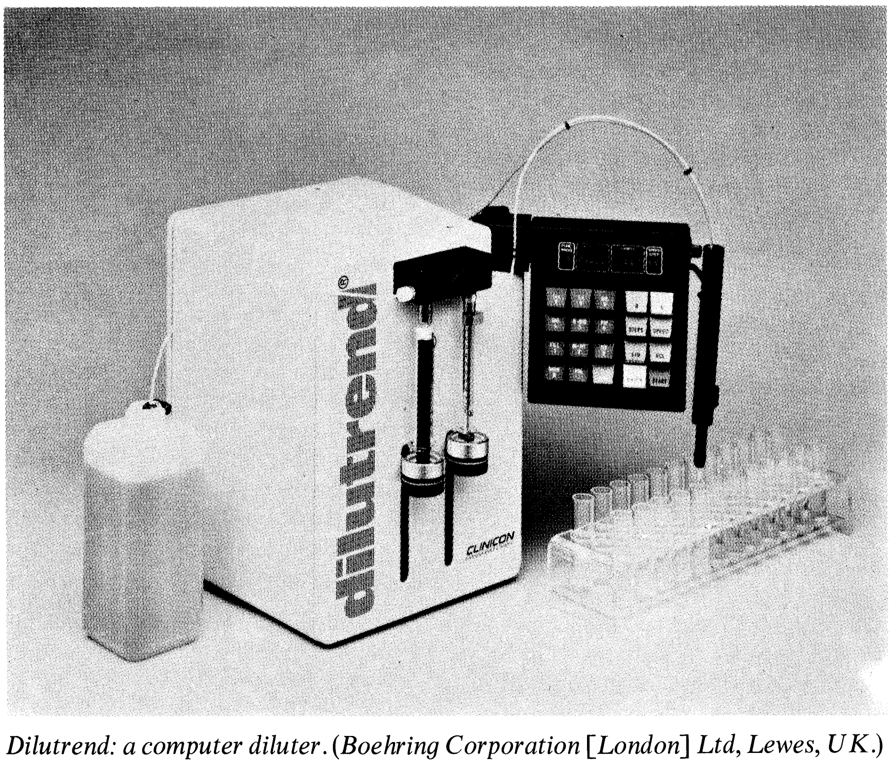

*Dilutrend: a computer diluter. (Boehring Corporation [London] Ltd, Lewes, UK.)*

### Programmable automatic deionizer

The first microprocessor-controlled, user-programmable automatic two-bed deionizer has been announced. The unit, Hydrofine 400, features counter-flow regeneration for high treated water purity and economy of regenerated chemicals; it provides treated water outputs in the range of 100–2500 l/h. The instrument is controlled by a solid-state electronic system incorporating a user-programmable microprocessor, with choice of regeneration by time clock, volume throughput or treated-water quality. Digital displays show time of day or volume of treated water and product resistivity, and the resistivity meter has an adjustable set point. An integral recirculation pump is a standard feature of the Hydrofine 400, it recycles treated water to the plant inlet maintaining a flow through the ion-exchange beds during periods of no demand, ensuring that there is no deterioration in treated water quality. Normal commercial-strength chemicals are used in the instrument so that no manual dilution is necessary. The unit can be readily duplexed for duty/standby operation.

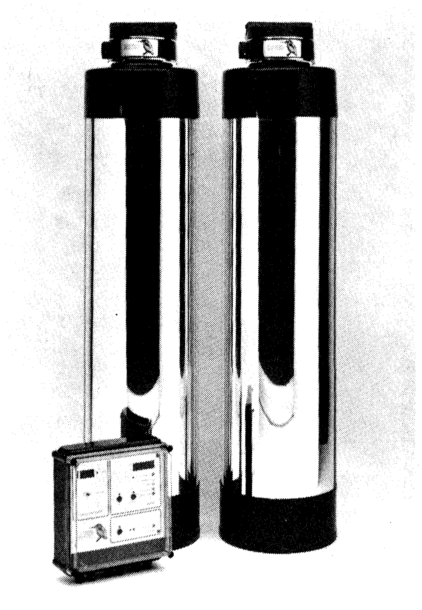

*The Chiltern Hydrofine 400 counter-flow regeneration automatic two-bed deionizer. (Chiltern Water Treatment Company Ltd, High Wycombe, UK.)*

*Further information about the unit can be obtained from Chiltern Water Treatment Company Ltd, PO Box 35, High Wycombe, Buckinghamshire HP11 1LK, UK. Tel.: 0494 446622.*

**Circle No. 14 on Reader Enquiry Card**

### Thin layer chromatography sample application

The CAMAG Automatic TLC Sampler I is a microprocessor-controlled device which offers automatic sample application in quantitative thin layer chromatography (TLC). Sample volumes can range from 100 nl to 20 $\mu$l and 16 samples can be handled at a time. Sample vials are placed in a tube rack and can be sealed with a membrane if necessary; then a fused silica capillary, connected to a stepping-motor operated dosage syringe, draws an appropriate quantity of sample solution from the vial and delivers the desired volume, at a pre-selected speed, to the TLC layer. The sequence in which samples are applied and their positioning on the layer can be selected. The capillary is automatically rinsed after each sample.

The Automatic TLC Sampler I is suitable for conventional and for high-performance TLC. Sixteen individual samples (for example, three calibration standards and five unknown, all in duplicate) can be applied in about 12 min.

*Further information from CAMAG, PO Box 34, CH 4132 Muttenz, Switzerland. Tel.: 061 613434.*

**Circle No. 15 on Reader Enquiry Card**

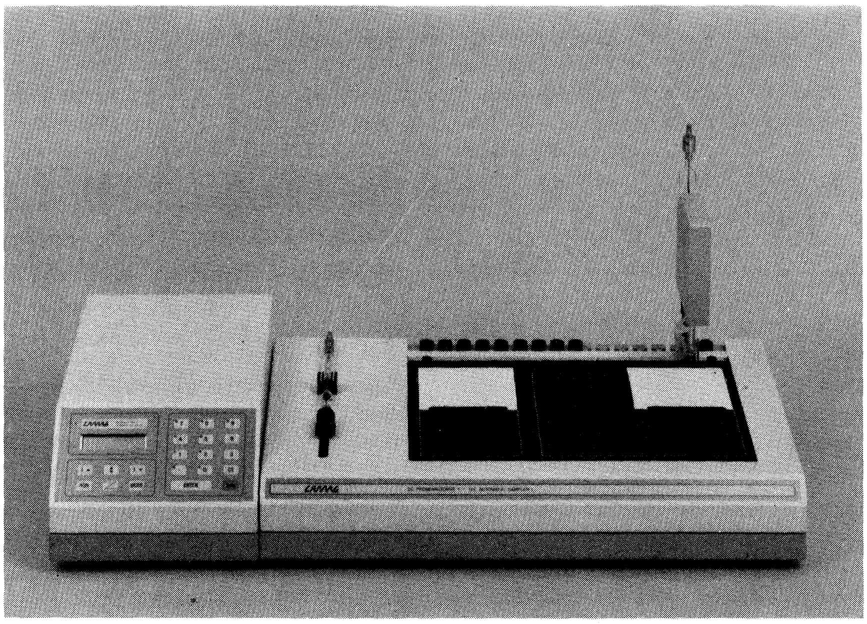

*CAMAG Automatic TLC Sampler I (CAMAG, Muttenz, Switzerland).*

### Coulometer from AERE Harwell

The Analytical R&D Unit at AERE Harwell have designed and are marketing a coulometry measurement system. The advantages of controlled potential coulometry are that a minimum of chemical operations is necessary (usually only a solution of the material in a dilute mineral acid is required); little sample-handling is needed (particularly valuable in the analysis of radio-active materials); that coulometry is an absolute technique; and the instrumentation can be calibrated against electrical standards.

The coulometry measurement system that Harwell are offering includes the following items: a small instrument crate complete with power-supplies and all necessary interconnecting cables; a coulometer module incorporating the potentiostat; a MOUSE (Microcomputer Organized for USErs) interface module which incorporates an auxiliary microprocessor and pulse-counting facilities; a Commodore microcomputer containing 32 kbytes of memory, and 80-column display screen, a keyboard and standard interface to provide overall control of the instrumentation and measurement procedure; a tape-cassette drive to store and load the computer programs; comprehensive general-purpose software (supplied on a tape cassette) which allows entry of input parameters, automatic control of the desired analyses, and subsequent display of the measurement results.

Optional extras are, for example, a printer, a range of special electrochemical cells, stepper motors, multiplexing facilities etc. Further, it is possible that Harwell will develop custom special-purpose software for the computer.

*For further information contact Dr J. Farren, Analytical R&D Unit, Instrumentation & Applied Physics Division, Building 148, AERE Harwell, Oxfordshire OX11 0RA, UK. Tel.: 0235 24141.*

**Circle No. 16 on Reader Enquiry Card**

### Thin-layer chromatography for aflatoxin analysis

A detailed description of a new method for the determination of aflatoxins ($B_1$, $B_2$, $G_1$ and $G_2$), is available free from CAMAG Chemie-Erzeugnisse und Adsorptionstechnik AG. Quantitative thin-layer chromatography is well-suited to analysis of food for aflatoxins. Using this technique, which involves parallel separations, a large number of samples can be analysed, time-consuming cleaning is unnecessary, and detection limits of between 25 and 50 ppt (parts per trillion) are better than legal requirements for determinations.

*Further information from CAMAG, Sonnenmattstrasse 11, CH 4132 Muttenz, Switzerland. Tel.: 061 613434.*

**Circle No. 17 on Reader Enquiry Card**

### Cuts in chromatography automation

All of the models in the SP 4100 Computing Integrator range have been reduced in price. The reductions average around 30% and are not part of a short-term offer, but, rather, are intended to reflect a genuine reduction in the cost of chromatographic automation. The standard SP 4100 will now cost £2395 (it was £3815) and the more powerful BASIC programming version of the instrument has been reduced from £4535 to £3550.

*Further information from Bernard Herd, Spectra-Physics Ltd, 17 Brick Knoll Park, St. Albans, Hertfordshire AL1 5UF, UK. Tel.: 0727 30131.*

**Circle No. 18 on Reader Enquiry Card**

### Laboratory micro

The SPI 80 is a microcomputer specifically designed for control and data-logging applications in laboratories. The machine has 64 k RAM, high-resolution graphics, twin floppy-disc drives and operates on disc-resident BASIC. It is possible to buy facilities for extra memory and for higher-level programming languages. The computer can also have a multi-user role linking several micros and giving rapid access to data acquired at different sites.

*Further information on the PSI 80 from Anaspec Research Laboratories Ltd, Pearl House, Bartholomew Street, Newbury, Berkshire, UK. Tel.: 0635 44329.*

**Circle No. 19 on Reader Enquiry Card**

### Reports on microprocessors in industry

A series of reports on microprocessors in industrial instrumentation is being produced by the Sira Institute. The reports, which cost £5·00 each, result from a programme initiated by Sira and supported by the UK Department of Industry's Electronics and Avionics Requirements Board, which aims to promote the UK development of LSI- and microprocessor-based instrumentation. The reports aim to disseminate, as widely as possible, practical guidance on the use of microelectronics in measurement and control equipment.

Three reports are now available:

The use of microprogrammable processors in industrial instrumentation, by D. Ibrahim (City University), in collaboration with W. H. Simmonds (Sira) and A. C. Davies (City University).

Production testing microprocessor-based instrumentation, by P. D. Britten and R. A. Brook (Sira).

An introduction to the IEC 625 (IEEE 488) standard interface, by J. C. Taunton (Sira).

Three more reports will be published shortly:

**Circle No. 25 on Reader Enquiry Card**

A guide to the tasks in developing microprocessor-based instruments and the resources required, by F. W. D. Woodhams (Sira).

A comparative study of the Intel 8086, Zilog 28000 and Motorola MC68000, by E. N. Goodyer and P. Neylan (Sira).

A guide to reliability aspects of microprocessor-based instrumentation development, by J. C. Taunton (Sira).

*Enquiries and orders to Miss M. A. Lee at Sira's Chislehurst Laboratories: Sira Institute Ltd, South Hill, Chislehurst, Kent BR7 5EH, UK. Tel.: 01 467 2636.*

**Circle No. 20 on Reader Enquiry Card**

### Gas chromatography autosampler

Perkin-Elmer have announced a computer-controlled autosampler for packed and capillary gas chromatography. Model AS-100B is designed for the SIGMA series of gas chromatographs; it has a pneumatically-operated injection system and an electronically-controlled sample tray which can hold up to 100 sample vials. The instrument is easy to use and the microprocessor allows the analyst to execute up to four different analysis programs during the sample sequence. Features of the autosampler include manual, automatic or remote control; variable analysis time from 1 to 999 min.; up to three injections per vial; sample number identification; use of microvials as well as standard 0·8 ml vials; variable flush facility. The AS-100B also has a variable injector dwell time so that complete reproducibility of analyses can be achieved even with difficult samples, for example high boiling or viscous compounds.

*Further information from Perkin-Elmer Ltd, Post Office Lane, Beaconsfield, Buckinghamshire HP9 1QA, UK. Tel.: 04946 6161.*

**Circle No. 21 on Reader Enquiry Card**

### Central gas control unit for gas chromatography

A gas-chromatography supply console has been built by Northern Technical and Chemical Services to a specification provided by Vauxhall Motors. The unit controls the three gases required for a pair of FI detectors used in the routine analysis of effluent and environmental samples. These tests are carried out around-the-clock so the design-brief specified features to minimize down-time, to increase safety in the event of a gas or

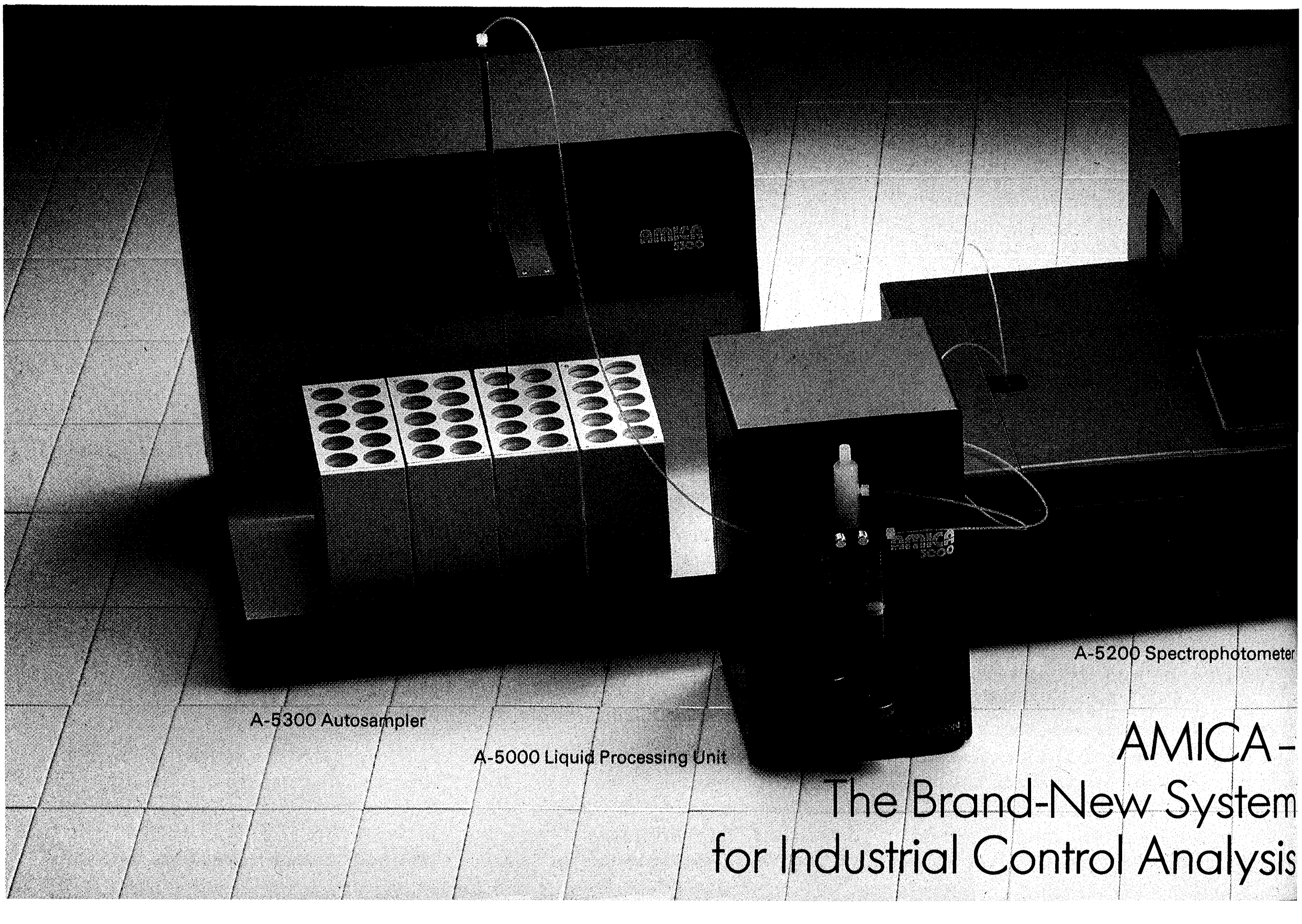

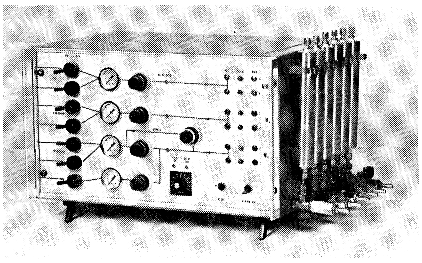

*Gas chromatography supply console for routine analysis of effluent and environment samples. (Northern Technical and Chemical Services, Liverpool, UK.)*

power failure and to permit safe close-down at a pre-selected time after working-hours. The console has three automatic cylinder change-over modules, which allow automatic change of gas cylinders without fluctuation in flow or pressure. Cylinder status and activity are indicated by neon signal lights. Although the automatic cylinder-change facility minimizes the risk of gas loss, the unit will cut-off all gases in the event of nitrogen failure and will also cut-off hydrogen on air failure and vice versa. The problem of power failure is overcome by the use of a rechargeable battery power-supply, which can be set via a delay timer to maintain operation for up to 2 h. A digital timeswitch can be set to close-down gas supplies at any time up to 24 h after setting. In this way a series of samples can be completed after-hours without operator intervention. The columns are protected during cool-down by a low-pressure nitrogen supply which is maintained at all times. Outlet gas supplies are divided into two streams for the two detectors and an extra, independently regulated nitrogen-line powers the pneumatic sample changer. Inlet feed-lines are routed through molecular-sieve driers to remove traces of water and other volatiles before entering the unit. Purge-valves are fitted to allow line clearance after renewal of cylinders.

*Further information about the unit can be obtained from NTC Services, 331 East-prescot Road, Liverpool L14 2DD, UK. Tel.: 051 228 4690.*

**Circle No. 22 on Reader Enquiry Card**

### Digital thermometer

Two casings are available for a new series of digital thermometer. The thermometer is calibrated for thermocouple and resistance thermometer sensors from below 0°C to 1100°C, with a switchable resolution of either 0·1°C or 1·0°C. The instrument is supplied in a plastic case as standard but a metal enclosure is available for applications where it is necessary to prevent ingress of dirt or moisture. The manufacturer also produces a range of manual probes so the thermometer can be used in laboratory work, for industrial and commercial applications—food processing for example.

*Further details from Industrial Instrument Services Ltd, Lynn Lane, Shenstone, Lichfield, Staffordshire WS14 0DY, UK. Tel.: 0543 480788.*

**Circle No. 23 on Reader Enquiry Card**

### 'Ion Chromatography in the Semiconductor Industry'

A pamphlet describing the role of their ion chromatographs in the determination of ionic contamination and device failure has been published by Dionex. The leaflet covers the specific application areas and features and benefits of ion chromatography, and summarizes four of the company's recently published aplication notes.

*'Ion Chromatography in the Semiconductor Industry' is available from Dionex (UK) Ltd, First Floor, The Parade, Frimley, Camberley, Surrey GU16 5HY. Tel.: 0276 29771; or Dionex Corporation, 1228 Titan Way, Sunnyvale, California 94086, USA. Tel.: 408 737 0700.*

**Circle No. 24 on Reader Enquiry Card**

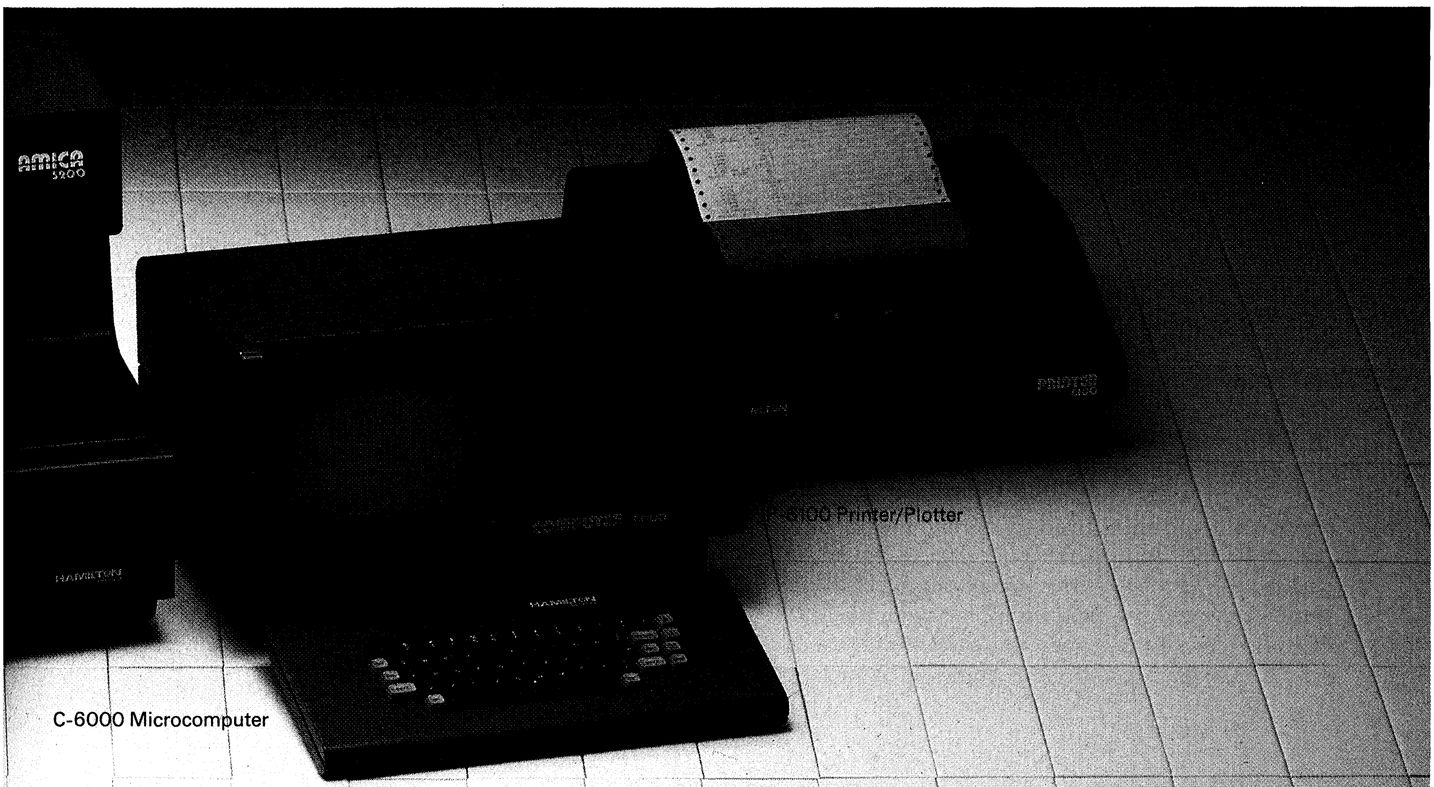

AMICA is an acronym meaning "Automated Modules for Industrial Control Analysis", an original concept for a new line of photometric and titrimetric analyzers.

### Sira multi-client project assessing programmable control systems

An assessment programme which will provide data to help companies select the best programmable control system for their particular application has been launched by the Sira Institute. The Institute is assessing systems that are widely available, medium priced and intended for general applications involving monitoring sequence control and loop control on plant and machinery. The evaluations will cover the following:

Definition of system capabilities in relation to requirements for logic control, sequence control, loop control, alarm, data logging and supervisory control.

Analysis of facilities for interfacing to plant and machinery.

Analysis of operator display and control facilities.

Analysis of communications capabilities for interfacing with computers and other systems.

Assessment of methods and facilities for system configuring and programming.

Evaluation of performance via benchmark tests of parameters such as response times, accuracy and capacity.

Assessment of safety, security, self-checking and diagnostic features.

Companies can obtain results of the assessments by joining a multi-client project which is already under way. Clients are involved in the selection of systems to be studied by Sira and also in what questions should be asked about them.

*Further information from W. H. Simmonds, Sira Institute Ltd, South Hill, Chislehurst, Kent BR7 5EH, UK. Tel.: 01 467 2636.*

**Circle No. 26 on Reader Enquiry Card**

### 'AutoAnalyst'

The January 1982 issue of the *AutoAnalyst* continues a series of short reports on Technicon's SMAC II system—discussing how the analytical processor has been redesigned so that it is easy to operate and maintain. The magazine also contains a summary of a paper on the Technicon H6000, an announcement of new LDM/SYSLAB software, and information on the RA-1000 system and Technicon's calibrators.

*Copies are available from F. V. Hooley, Technicon Instruments Co. Ltd, Evans House, Hamilton Close, Basingstoke, Hampshire, UK. Tel.: 0256 29181.*

**Circle No. 27 on Reader Enquiry Card**

### New UV/VIS single-beam spectrophotometers

The LKB Ultrospec 4050 (200–900 nm) and 4051 (325–900 nm) are single-beam spectrophotometers which have a unique microprocessor-based optical system. The instruments have a single control panel and are operated with eight smooth keys. The cell compartments are big enough to accommodate six 10 mm cells or four cells up to 50 mm path-length. Wavelength, measurement value, and cell numbers are shown on digital displays; there is also an LED indication of lamp(s) in use and mode of operation—absorbance, transmission or concentration. The microprocessor automatically verifies the wavelength accuracy of the instruments within 90 s from switch-on, and if necessary the Ultrospec is re-calibrated. In the UV model (Ultrospec 4050) the optical system incorporates deuterium and tungsten halogen lamps, and the computer optimizes energy throughput according to the working wavelength. Mechanical lamp changes are eliminated, together with the need to remember to change sources. A single solid-state detector operates over the whole wavelength range—again eliminating a selection control. The dark current from the detector is constantly monitored and corrected, so very accurate measurements can be made at high absorbance levels.

The traditional criteria of high performance—low stray light, base-line stability, low noise—match or improve on other instruments on the market.

Accessories include a wide range of cell holders; the LKB Autofill, which offers rapid sample handling of volumes around 500 μl; and a choice of recorders and digital printers.

*Further information from Phil Morris, LKB Instruments Ltd, 232 Addington Road, Selsdon, South Croydon CR2 8YD, UK. Tel.: 01 657 8822.*

**Circle No. 28 on Reader Enquiry Card**

### Chemical auto-analyser control system

A microprocessor control unit for chemical analysers, called ARCAMS, has been further developed so that it can be used on 12/60, 12-channel systems. ARCAMS was designed by British biochemists and is already used in several UK hospitals. The benefits of adding the control unit to an existing installation include savings in reagent costs, increased sampling speed, elimination of manual phasing, facilities for carry-over and drift correction.

*A comprehensive brochure about ARCAMS is available from Jeff Young, Advanced Medical Supplies Ltd, 19 Holder Road, North Lane Industrial Estate, Aldershot, Hampshire, UK. Tel.: 0252 317016.*

**Circle No. 29 on Reader Enquiry Card**

